# QuickStats

**Published:** 2014-02-28

**Authors:** FROM THE NATIONAL CENTER FOR HEALTH STATISTICS

**Figure:**
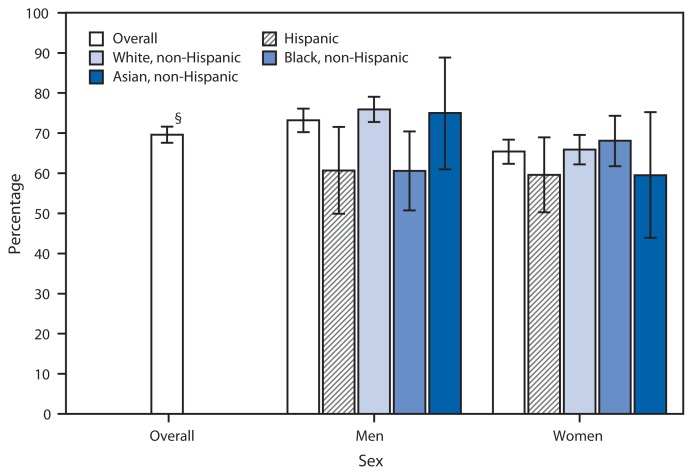
Percentage of Adults Aged ≥40 Years Who Ever Had a Cardiovascular Event^*^ and Are Now Taking Low-Dose Aspirin to Prevent or Control Heart Disease, by Sex and Race/Ethnicity — National Health Interview Survey, 2012^†^ ^*^ Includes heart attack (myocardial infarction), angina pectoris, coronary heart disease, or stroke. ^†^Estimates are based on household interviews of a sample of the noninstitutionalized U.S. civilian population and are derived from the National Health Interview Survey sample adult component. ^§^95% confidence interval.

In 2012, 69.6% of adults aged ≥40 years who ever had a cardiovascular event (73.2% of men and 65.4% of women) were taking low-dose aspirin to prevent or control heart disease. Non-Hispanic white men (75.9%) were more likely to be taking low-dose aspirin compared with Hispanic (60.7%) and non-Hispanic black men (60.6%). No statistically significant differences were oberved among women by race/ethnicity.

**Source:** National Health Interview Survey, 2012 data. Available at http://www.cdc.gov/nchs/nhis.htm.

**Reported by:** Renee M. Gindi, PhD, iuz2@cdc.gov, 301-458-4502; Brian W. Ward, PhD.

